# Acidic and Electrosurface Properties of Binary TiO_2_-SiO_2_ Xerogels Using EPR of pH-Sensitive Nitroxides

**DOI:** 10.3390/gels7030119

**Published:** 2021-08-11

**Authors:** Denis O. Antonov, Daria P. Tambasova, Andrey B. Shishmakov, Igor A. Kirilyuk, Elena G. Kovaleva

**Affiliations:** 1 Institute of Chemical Engineering, Ural Federal University Named after the First President of Russia B N Yeltsin, Mira St., 19, 620002 Yekaterinburg, Russia; d.p.tambasova@urfu.ru (D.P.T.); e.g.kovaleva@urfu.ru (E.G.K.); 2 Laboratory of Organic Materials, I. Ya. Postovsky Institute of Organic Synthesis, Ural Branch of the Russian Academy of Sciences, Akademicheskaya/S. Kovalevskoy, 22/20, 620990 Ekaterinburg, Russia; Mikushina@ios.uran.ru; 3 Institute of Organic Chemistry, Siberian Branch of the Russian Academy of Sciences, Akad. Lavrent’ev Av. 9, 630090 Novosibirsk, Russia; kirilyuk@nioch.nsc.ru

**Keywords:** xerogels characterization, TiO_2_-SiO_2_, surface electrostatic potential, electron paramagnetic resonance, pH-sensitive nitroxide radicals, titration curve

## Abstract

The binary xerogels TiO_2_-SiO_2_ are widely used as catalysts and their carriers in organic synthesis. Characterization and adjustment of the electrostatic properties of the surface and the local acidity inside the pores, are necessary for the further development of TiO_2_-SiO_2_ xerogels applications. This research investigates acid–base equilibria in the pores, and the surface electrostatic potential (SEP) of binary TiO_2_-SiO_2_ xerogels, by the EPR of stable pH-sensitive nitroxide radicals. These radicals are small enough to penetrate directly into the pores, and to be adsorbed onto the surface of the material under study. This makes it possible to obtain valuable information on the acidic and electrosurface properties of the studied system. The highest negative surface electrical charge associated with surface electrical potential (SEP) was equal to −196 ± 6 mV. It was induced by the surface of the sample with a 7% TiO_2_ content. The local acidity inside the pores of this sample was found to be higher, by approximately 1.49 pH units, as compared to that in the external bulk solution.

## 1. Introduction

The binary SiO_2_-TiO_2_ xerogels have been applied in a variety of fields of science and technology. These materials are widely used in optical waveguides and sensors [1], hydrophilic coatings, selective sorbents for purification of air [2,3], and aqueous media [4] made from organic and inorganic compounds [5,6,7], and in self-purifying materials, based on glass [8] or cotton [9]. They have been successfully applied as carriers of catalysts in the Fischer–Tropsch process [10] and various oxidation reactions [11], as well as a matrix for the immobilization of the biomacromolecules and components of drug delivery systems [12]. Cu(II)-containing TiO_2_-SiO_2_ xerogels were investigated and found to be efficient photocatalysts [13]. Polyfunctional heterogeneous systems, based on Cu(II)-containing titanium dioxide, were proven to consist of various self-organized copper-containing structures, which are catalytically active in the reactions of hydroxyarene oxidation [14,15,16,17].

At present, the sol-gel technology is widely used as a method of synthesis, based on the reactions that were developed by Nikolaon and Teichner in 1976, to obtain silica aerogels [18]. These are the reactions of the hydrolysis and gelation of alkoxides or other reactive compounds in an alcohol solution, with the addition of acids and alkalis as catalysts.

It was found that in different processes utilizing these xerogels, the acidity of the solutions contacting with them plays an important role; however, the pH values of the external solution (*pH_ext_*) can differ from that of the solution inside pores (*pH_loc_*). The reasons for these differences can be the changes both in the intrinsic electrical charge of the surface and in the properties of the surface water.

The acidity and electrosurface properties of the surface are determined by the number and type of functional groups of the materials, and depend on the synthesis conditions and phase composition [19]. The surface of silica were found to have five types of functional groups, in different proportions [20].

Previous relevant studies have shown that the classical methods of surface acidity measurement are mostly based on the spectrophotometric studies of adsorbed dyes (Hammett indicators) during titration, or the measurements of microcalorimetry and the temperature-programmed desorption of other basic and/or acidic probes of various strengths, to estimate the concentration and strength of acidic sites [19,21,22]. Several spectroscopic techniques, including conventional infrared (IR) and Fourier transform infrared (FTIR) spectroscopy, magic angle rotation NMR and electron paramagnetic resonance (EPR), ultraviolet (UV), visible and photoluminescence spectroscopy, Raman scattering, and X-ray radiation photoelectron spectroscopy, have been used effectively for the identification of some catalytic mesoporous materials and the characterization of the acid–base properties of different surface locations [11,23].

These spectroscopic methods have their advantages and disadvantages. EPR spectroscopy of ionizable nitroxides is especially useful in the investigation of opaque or turbid materials [24,25,26]. The EPR of spin probes exhibits better concentration sensitivity compared to solid-state NMR. The EPR method is based on the spectroscopic observation of the reversible protonation of stable free radicals, e.g., pH-sensitive nitroxide radicals (NR) [27] or triarylmethyl radicals [28].

Electron paramagnetic resonance (EPR) is a physical phenomenon that is observed in molecular systems possessing unpaired electronic spins. This phenomenon involves the absorption of electromagnetic radiation of a frequency that is found to be in resonance with the gyroscopic precession of the spins’ magnetic moments, occurring in a static magnetic field. The resonance condition can be reached by scanning either the magnetic field or frequency; technical reasons make it easier to sweep the magnetic field. The most common way to detect EPR is the continuous wave (CW) EPR experiment, in which the magnetic field is changed, while the frequency is kept constant. EPR spectroscopy is a versatile and exceptionally sensitive technique for detecting and studying molecular systems possessing unpaired electronic spins [25]. The scope of EPR applications in analytical chemistry can be significantly expanded by using spin labels and spin probes, that is, paramagnetic species introduced to gain information on the system. To be suitable for analytical applications, spin labels and probes should immediately report on the properties of interest, produce ERP signals that are distinguishable from the background, and leave the system only minimally perturbed by their presence [25]. The EPR spectroscopy of pH-sensitive spin probes is based on the observation of nitroxide radicals that contain basic nitrogen functionalities in the heterocyclic ring. The protonation of these functionalities changes the intermolecular electric field, which, in turn, affects the nitroxide magnetic parameters, such as nitrogen hyperfine coupling A-tensor and *g*-factor matrix, as reflected by the nitroxide CW EPR spectra [27]. These phenomena make the EPR spectra of such nitroxides exceptionally sensitive to local *pH* (*pH_loc_*) values [28]. As the method is based on EPR detection, it is fully applicable to opaque and not transparent samples, such as the nano and mesoporous materials that are studied in this research.

The general objective of this work was to investigate the surface electrostatic potential (SEP), surface charge, and *pH_loc_* of the binary intrinsic and annealed TiO_2_-SiO_2_ systems with different titanium contents, using continuous wave (CW) X-band (9 GHz) EPR and a pH-sensitive nitroxide radical (4-dimethylamino-2-ethyl-5,5-dimethyl-2-(pyridine-4-yl)-2,5-dihydro-1H-imidazol-1-oxyl) as a spin probe.

## 2. Results and Discussion

### 2.1. Xerogels Characterization

**Figure 1:**
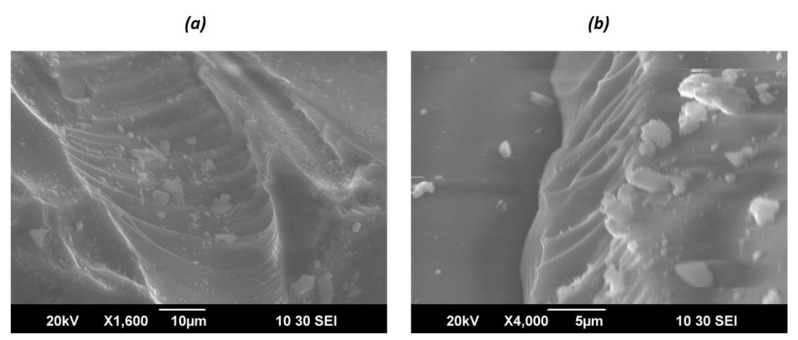
SEM image of TiO_2_ (7%)-SiO_2_ samples. Metric scale (**a**) 10 μm and (**b**) 5 μm in 1 cm of the image.

The morphology of the oxide xerogels was examined by SEM. Figure 1 shows the SEM micrographs of the samples TiO_2_ (7%)-SiO_2_. The images demonstrate porous structure synthesis samples.

**Figure 2:**
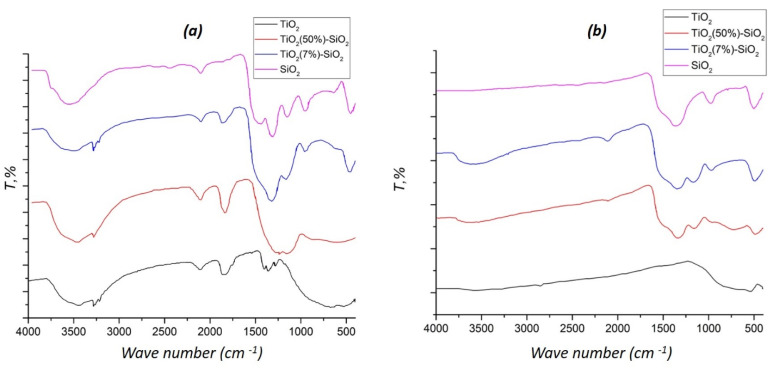
FT-IR spectra of synthesized xerogels (**a**) before and (**b**) after annealing at 850 °C.

The infrared spectrum of the SiO_2_ xerogel (Figure 2a) had an intense complex-shaped absorption band, with the maxima at 1155 and 1062 cm^−1^, and an absorption band with a maximum at 798 cm^−1^ corresponding to stretching vibrations of Si–O–Si bonds.

The complex-shaped band corresponded to asymmetric stretching vibrations and the average intensity band at 798 cm^−1^ to symmetric stretching vibrations of SiO_4_ tetrahedra. The absorption band with a maximum at 945 cm^−1^ demonstrated symmetric stretching vibrations of nonbridging Si–O– bonds. An intense band at 435 cm^−1^ corresponded to the bending vibrations of polyhedra. The absorption bands of stretching vibrations of surface hydroxyl groups and water appeared to be in the range of 3700–3000 cm^−1^. A broadband of average intensity in the region of 1626 cm^−1^ corresponded to the bending vibrations of water [29]. The ratios of the intensities of the absorption bands of Ti–O–Si and Si–O–Si groups at 950 cm^−1^ and 1079 cm^−1^, respectively, for binary oxide xerogels as a function of the oxide xerogels composition are shown in Table 1. These data demonstrated that the number of Ti–O–Si groups decreased with increasing titanium oxide fraction of the binary system.

Annealing at 850 °C (Figure 2b) caused dehydroxylation of the SiO_2_ sample, as evidenced by the absence of absorption bands at 3700–3000 cm^−1^, and the non-bridging Si−O−bonds in the spectra. Similarly, in titanium dioxide, due to calcination, the absorption bands of the Ti−OH groups (1126, 1095, and 1036 cm^−1^) were also not found. In the spectra of binary xerogels and TiO_2_, water absorption bands (3700–3000 cm^−1^, 1625 cm^−1^) were present, which was the result of sorption from the atmosphere, due to the hygroscopicity of the samples.

### 2.2. Rotational Motion of Spin Probes in Individual Oxides and Binary Oxide Xerogels

The acid-base characteristics of water in the micropores of inorganic oxides and ion-exchange resins, are known to differ from those in the external solution [30]. To measure the *pH_loc_* inside the pores of the samples, the pH-sensitive spin-probe technique was used [24]. We evaluated the SEP of individual and binary oxide xerogels, and its effect on the acidity of the solution inside the pores in the samples.

**Figure 3:**
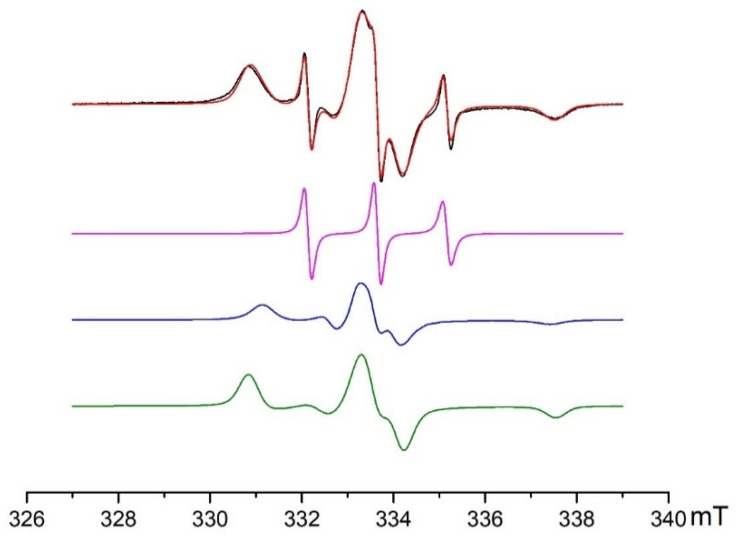
Experimental (black curve) and simulated (red curve) EPR spectra of the NR on the surface of the TiO_2_ (7%)-SiO_2_ binary system at *pH_ext_* = 5.58. The simulated EPR spectrum of the NR is a superposition of three signals, namely, fast-motioned NR (**magenta curve**), slow-motioned NR in the protonated (**blue curve**), and non-protonated forms (**green curve**).

At a *pH_ext_* below 10, the experimental EPR spectra of NR in the TiO_2_-SiO_2_ and SiO_2_ samples represented a superposition of isotropic and anisotropic signals (Figure 3), indicating the coexistence of two forms of NR, with different molecular mobilities. The isotropic signal (Figure 3, highlighted in magenta) corresponds to the fast-motioned NR molecules that are located in a diffusion layer inside the pores [31]. The signals produced by the NR molecules allow information on the acidity of the solution inside the pores of oxide materials to be obtained [30,32]. The anisotropic signal (Figure 3, highlighted in blue and green) was generated by the NR molecules that were adsorbed onto the surface. This signal provides information on SEP [33].

At a *pH_ext_* value greater than 10, only the isotropic EPR signal characteristic of fast-motioned NR, which was located inside the pores of the oxide xerogels, was recorded.

The NR that was adsorbed on the TiO_2_ surface only gave isotropic spectra for all the *pH_ext_* values.

### 2.3. EPR Titration of NR in the Individual and Binary Intrinsic Oxides

**Figure 4:**
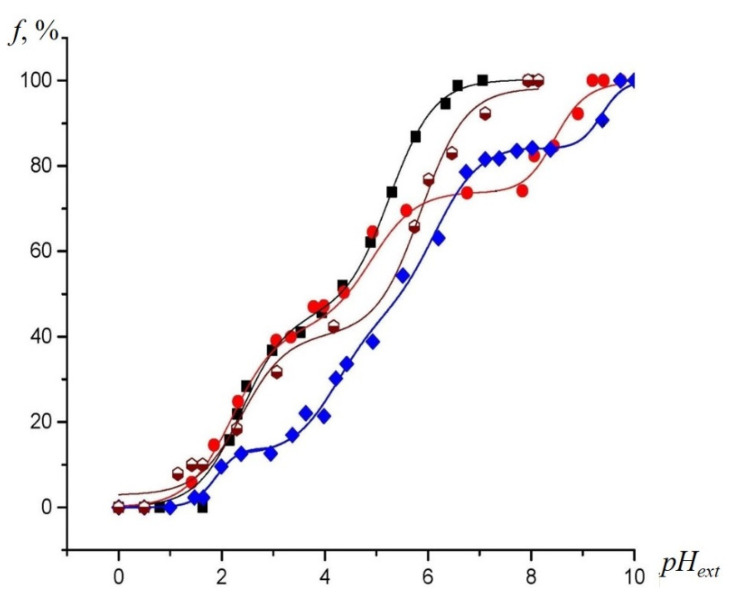
Experimental EPR titration curves for the NR at 293 K obtained by plotting the fast- and slow-motioned component of the EPR spectra of the NR in the non-protonated form (*f*) vs. *pH_ext_* in aqueous solution (**black squares**), and inside the SiO_2_ (**red circles),** TiO_2_ (50%)-SiO_2_, (**brown hexagons**) and TiO_2_ (7%)-SiO_2_, (**blue diamonds**), respectively.

EPR titration curves of the NR in external aqueous solution, as well as in SiO_2_ and binary TiO_2_-SiO_2_ xerogels, are shown in Figure 4. For NR in external aqueous solution, the isotropic hyperfine splitting constant (HFS) was plotted vs. the *pH_ext_*. The fraction of the slow-motion component of NR inside the pores was determined via EPR spectra simulation, and was used as a pH-dependent parameter for the investigation of SiO_2_ and binary TiO_2_-SiO_2_ xerogels.

The upper part of the titration curve for the SiO_2_ sample was found to be shifted to the right (to the higher *pH_ext_* values), relatively to the EPR titration curves of the NR in external aqueous solution (the black curve), which indicated a negative charge of the silicon dioxide surface in a neutral and weakly acidic solution (at *pH_ext_* > 3–4) [30,33]. As the value of *pH_ext_* decreased, the EPR titration curve for this oxide came closer to the calibration curve and intersected it, thus shifting to the left, relatively to that at a *pH_ext_* less than three. It is known that the hydroxylated silica surface has point of zero charges (*PZC*) at *pH_ext_* values between two and three, and becomes charged positively at the lesser value *pH_ext_* [34].

The horizontal plateaus in the titration curves of the NR inside SiO_2_, and the TiO_2_-SiO_2_ binary xerogels obtained by simulating the anisotropic spectra, reflected the invariance of the fraction of the non-protonated form of the NR, *f*, with a change in *pH_ext_*. These plateaus were explained by the ionization of the silicon dioxide silanol groups [33]. The *pKa* values of functional groups can be measured by projecting any pH-sensitive parameter of an EPR spectrum of the NR inside the material corresponding to a horizontal plateau on the calibration curve, and then to the *pH_ext_* axis [33]. In our case, the pH-sensitive parameter was *f* (Figure 4).

For the binary TiO_2_ (7%)-SiO_2_ samples, the EPR titration curves of the NR showed a greater shift to the right (to the higher *pH_ext_* values) from the calibration curve, as compared to the curve for the individual SiO_2_, indicating the stronger negative charge of the pores surface at high values of *pH_ext_*. The existence of a plateau in the *pH_ext_* range of 2.5–3.5 indicated the formation of new acidic centers with a higher acidity than that of the silanol groups in the individual SiO_2_. As a result of the deprotonation of these groups at higher *pH_ext_* values, the surface acquired a negative charge (SEP). This charge caused a decrease in the acidity of the remaining silanol groups, shifting the plateau in the upper part of the titration curve further upwards (to the higher *pH_ext_* region). Remarkably, the titration of the TiO_2_ (50%)-SiO_2_ sample showed a much lower shift of the titration curve to the right (the surface became charged negatively). We noted that a buildup of the titanium dioxide content in the samples led to an increase in the dissociation constant of the silanol groups, since the upper plateau on the titration curve for NR in the TiO_2_ (7%)-SiO_2_ sample was positioned above that for pristine SiO_2_. We assumed that for the TiO_2_ (50%)-SiO_2_ sample, the titration of silanol groups would take place at the *pH_ext_* values above the sensitivity range of the NR used. Therefore, we did not observe the characteristic plateau on the titration curve for this sample. Interestingly, the EPR titration curve of the NR was shifted higher to the right for a binary system with a 7% mol of TiO_2_ content. The IR spectroscopy data (Table 1), which indicated the maximum amount of the mixed phase for this sample, allowed us to conclude that an increase in the negative surface charge for this sample is associated precisely with the formation of a mixed Ti–O–Si phase.

The plateau in the titration curves of the NR in binary oxides in the *pH_ext_* value range from 2.5 to 4.5, corresponds to the deprotonation of either the silanol groups of the silica located near titanium atoms or new functional groups arising in the mixed Ti–O–Si phase. It proved the presence of the mixed Ti–O–Si phase, along with the individual phases of SiO_2_ and TiO_2_ in the binary TiO_2_-SiO_2_ xerogels, by the NMR method, and it was noted that the functional groups that were associated with the mixed-phase showed higher acidity than the silanol ones [35]. These groups, with similar values of *pKa*, were revealed in the study of binary ZrO_2_-SiO_2_ oxide xerogels, which indicated that they (groups) belonged to a mixed phase [36].

### 2.4. EPR Titration of NR in the Individual and Binary Annealed Oxides

The annealing of SiO_2_ samples at 850 °C led to the formation of multiple acidic centers on the sample surface, and resulted in the shift of the titration curve to the right at a *pH_ext_* value range of 4–5.5. At the higher *pH_ext_* values, these centers were ionized, providing the negative SEP, which in turn decreased the acidity of the remaining silanol groups. As a result, further ionization of these silanol groups occurs at the higher *pH_ext_* values, with the titration curve plateau shifting upwards. We observed that the shift of the titration curve for the NR in the annealed sample at *pH_ext_* > 8, was found to be lesser than that for the sample before annealing. This effect was due to the partial decomposition of acidic silanol groups, which led to a decrease in the total negative charge of the surface (SEP) at alkaline *pH_ext_*.

**Figure 5:**
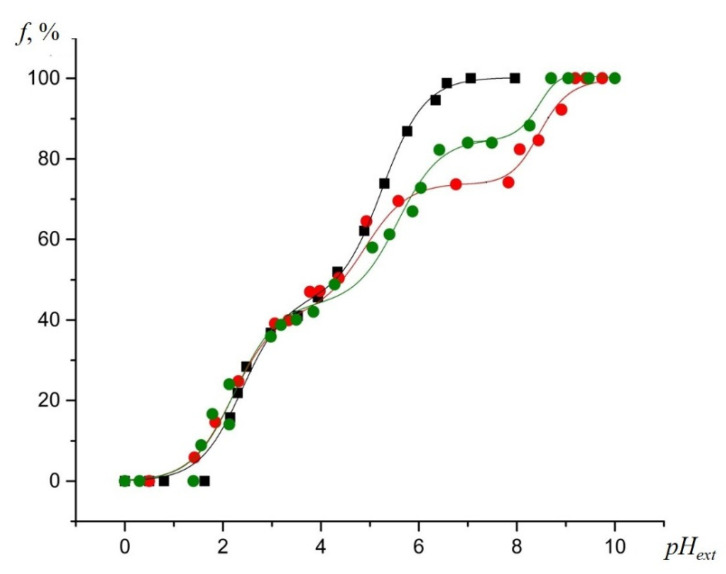
Experimental EPR titration curves for the NR at 293 K obtained by plotting the fast- and slow-motioned component of the EPR spectra of the NR in the non-protonated form (*f*) vs. *pH_ext_* in aqueous solution (**black squares**) and inside the SiO_2_ (red circles), SiO_2_ annealed at 850 °C (**green circles),** respectively.

Specifically, the increase in the *pKa* value of silanol functional groups, after annealing at 850 °C, was attributed to the fact that upon completion of the titration in the annealed sample, SEP became greater (in absolute value) than that for the unannealed one (Figure 5, green curve). Within the *pH_ext_* range from four to seven, the curve is positioned at the right, relatively to the red curve. Hence, the dissociation of silanol groups (removal of the positively charged hydrogen H^+^ ions) in these samples was impeded not only by the bond strength in the acid group Si–O–H, but also by the increased negative charge of the entire matrix of the sample. An increase in the negative charge of the silicon oxide surface after calcination can be associated with the formation of both siloxane groups on the silicon oxide surface and Lewis acid sites. This is due to the detachment of silanol groups during heat treatment.

**Figure 6:**
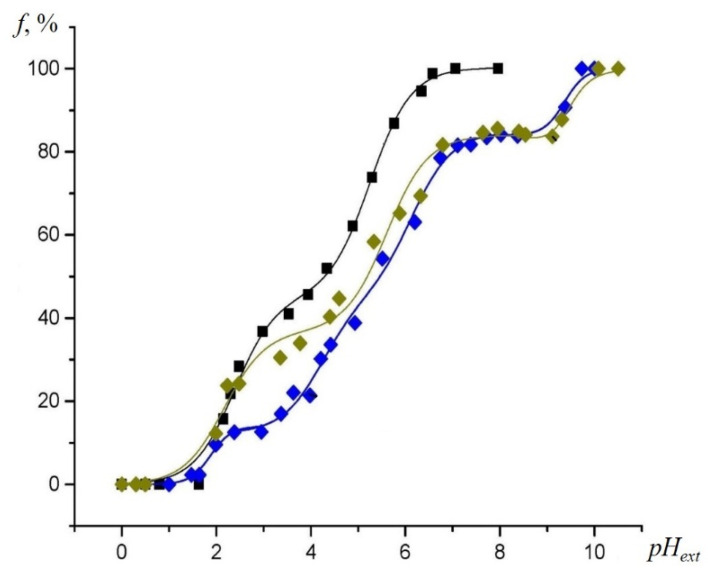
Experimental EPR titration curves for the NR at 293 K were obtained by plotting the fast- and slow-motioned component of the EPR spectra of the NR in the non-protonated form (*f*) vs. *pH_ext_* in aqueous solution (**black squares**), and inside the TiO_2_ (7%)-SiO_2_ (**blue diamonds**), TiO_2_ (7%)-SiO_2_ annealed at 850 °C (**green diamonds**), respectively.

In contrast, annealing of the binary TiO_2_-SiO_2_ samples at 850 °C did not lead to a decrease in the total number of ionized groups at alkaline *pH_ext_* values (Figure 6). Thus, the TiO_2_ additive increased the thermal stability of the silanol groups. However, strongly acidic groups present only in binary oxides, and providing a plateau in the titration curve at pH 2–4 completely disappeared from the sample surface. Acidic centers that are similar to those observed in the annealed SiO_2_ were formed, instead of exhibiting on the titration curve plateau at the *pH_ext_* value of 2.5–4.5. The dissociation constants of the functional groups before and after calcination were found to be equal to 1.90 ± 0.08 and 2.90 ± 0.08, respectively.

A broad singlet in the EPR spectra of the NR appeared only for the annealed TiO_2_ (50%)-SiO_2_ sample. This occurred due to the presence of coal that formed as a result of burning the organic fraction of the precursors retained in the sample pores [29].

### 2.5. Effect of Incorporation of Cu(II) Ions into the Binary Oxides on EPR Titration of NR for Cu(II)-Containing Binary Xerogels

**Figure 7:**
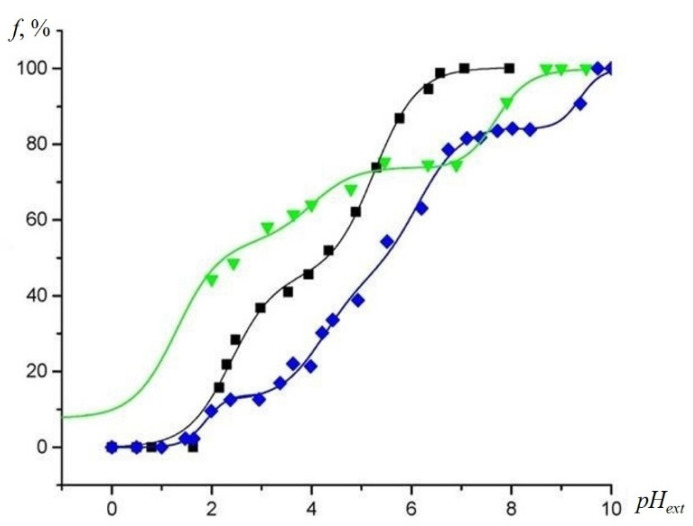
Experimental EPR titration curves for the NR at 293 K obtained by plotting the fast- and slow-motioned component of the EPR spectra of the NR in the non-protonated form (*f*) vs. *pH_ext_* in aqueous solution (**black squares**) and inside the TiO_2_ (7%)-SiO_2_ (**blue diamonds**), TiO_2_-SiO_2_-Cu(II) (**green triangles**), respectively.

The incorporation of Cu(II) into TiO_2_-SiO_2_ samples allowed partial compensation for the negative charge, with a pronounced shift of the titration curve to the left, as shown in Figure 7. The dissociation constants of the functional groups (*pKa* values) of different oxide xerogels, and the highest SEP of their surface (in absolute value), are shown in Table 2. The decrease in the *pKa* can be associated with the decline in the negative charge of the sample matrix. It facilitated the dissociation of the silanol groups, and, as a result, the plateau in the titration curve was shifted down to the lower *pH_ext_* values.

**Figure 8:**
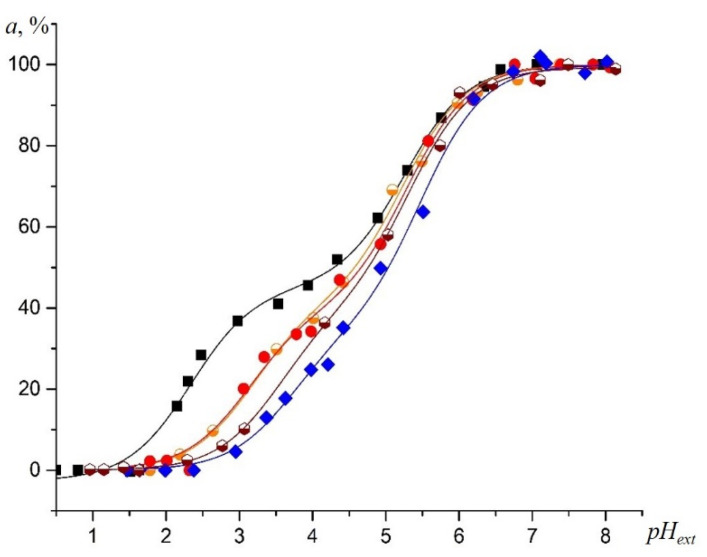
Experimental room-temperature EPR titration curves for the NR obtained by plotting isotropic nitrogen HFS, *a* vs. *pH_ext_* at ionic strength *I* = 0.1 M (**black squares**) and reporting on the average acidity inside the pores of TiO_2_ (**orange circles**), SiO_2_ (**red circles**), TiO_2_ (7%)-SiO_2_ (**blue diamonds**), TiO_2_ (50%)-SiO_2_ (**wine hexagons**).

As was mentioned above, no signal in the EPR spectrum for immobilized NR was observed in the TiO_2_ samples. However, titration of the fast-motioned NR molecules near the TiO_2_ and SiO_2_ surfaces led to similar *a,%* vs. *pH_ext_* dependences (Figure 8) *(a* is the pH-sensitive parameter, characterizing the hyperfine coupling constant, HFS constant).

The EPR titration curves for the dissolved NR near the surface of the SiO_2_ samples were significantly shifted to the right, relatively to the titration curve for the NR in the bulk aqueous solution. The shift is more noticeable at a *pH_ext_* less than 4.5, which can be ascribed to an increased acidity of the solution inside the samples’ pores, compared to the *pH_ext_* [30], due to the surface charge (close to zero) of *pH_ext_* (Figure 4). The highest shift of the EPR titration curves for the NR to the right, relatively to that in the bulk aqueous solution, was observed in the TiO_2_ (7%)-SiO_2_ sample (Figure 4 and Figure 8). The surface charge affects the acid–base equilibrium of the NR in a solution inside the pores.

The differences between *pH_ext_* and *pH_loc_* in the oxide xerogels are shown in Table 3.

## 3. Conclusions

CW X-band EPR spectroscopy of a pH-sensitive nitroxide radical, used as a free-motioned or adsorbed spin probe, was applied to study acid–base equilibria in the pores and surfaces of TiO_2_-SiO_2_ binary xerogels that were prepared by a sol-gel technique in the wet ammonia vapors atmosphere. The results showed that the variation in the composition of binary xerogels led to a change in both the electrosurface and acidic properties of the materials studied.

The new acidic centers, with a higher acidity than silanol groups, were formed in the binary system TiO_2_ (7%)-SiO_2_. Moreover, these samples showed a higher content of acidic groups on the surface, hence, the higher negative surface electrical potential compared to the pure silica samples (−196 to −204 mV vs. −137 to −159 mV). The local acidity inside the sample’s pores was found to increase by approximately 1.49 pH units, as compared to the bulk external solution. The annealing of pure silicon oxide at 850 °C decreased the number of silanol groups on the surface and increased the value of the dissociation constant of these groups. While the calcination of the TiO_2_ (7%)-SiO_2_ sample did not affect a decrease in the number of silanol groups, the breakdown of strongly acidic groups in the substituted oxide was observed.

## 4. Materials and Methods

### 4.1. Materials

Tetraethoxysilane 98% (TEOS), tetrabutoxytitanium 99% (TBT) precursors were purchased from Alfa Aesar (USA) and used without further purification.

The stable nitroxide radical 4-dimethylamino-2-ethyl-5,5-dimethyl-2-(pyridine-4-yl)-2,5-dihydro-1H-imidazol-1-oxyl used as a pH-sensitive spin probe, was synthesized according to previously described protocol [27].

### 4.2. Synthesis of Binary TiO_2_-SiO_2_ Xerogels

The synthesis of the samples was carried out by the sol-gel technique, according to the protocol previously described in [29], using titanium and silicon alkoxides as precursors. The hydrolysis of the samples was performed in presence of wet ammonia vapors.

Samples with 7% and 50% TiO_2_ were obtained by mixing TBT and TEOS in ratios of 1:9 and 6:4, respectively. The synthesis was carried out in porcelain dishes with a round bottom, 60 mm in diameter and 30 mL in volume. To synthesize the SiO_2_ and TiO_2_ individual oxides, 10 mL of TBT or TEOS were subjected to hydrolysis. The dish was placed into a 3 L desiccator containing a beaker with 100 mL of 10% aqueous ammonia and stored until hydrolysis was complete. After hydrolysis, the samples were dried for 48 h at room temperature, then kept in a drying oven at 90 °C for 24 h. Some samples were calcined annealed in a quartz reactor at a temperature of 850 °C (heating rate 10 °C/min) in air flow with a rate of 0.075 m^3^/h for an hour.

The TiO_2_ (7%)-SiO_2_-Cu (II) samples were obtained by dissolving 0.01 g of CuCl_2_ × 2H_2_O in the appropriate TEOS/TBT mixture with a subsequent treatment, according to the protocol above.

Compact FT-IR spectrometer: ALPHA II (manufacturer: Bruker Optics) was used for FT-IR measurements in the wavenumber range from 4000 to 400 cm^−1^. Scanning electron microscope (SEM) images were obtained by using a Carl Zeiss EVO LS 10 device (manufacturer: Carl Zeiss NTS, Jena, Germany).

### 4.3. EPR-Based Spin Probe Method for Measuring Acid–Base and Electrosurface Properties

Potentiometric titration of powdered individual and binary oxides in the range of *pH_ext_* from 1 to 10 was carried out by a conventional batch multi-sample technique [30,37]. Titration was carried out at 293 K with 0.1 N solutions of acid (HCl) and alkali (NaOH).

The samples were kept in the NR solution with a concentration of 1 × 10^−4^ M for 2 h to establish dynamic equilibrium between the external bulk solution and the solution inside solid phase. Thereafter, pH values of the equilibrium *pH_ext_* were measured by a 3-in-1 combination pH electrode and a Mettler Toledo FP20 pH meter (Mettler-Toledo, Columbus, OH, USA) with the specified accuracy of ±0.01 pH. The ±0.01 pH error was significantly less than typical errors of ±0.05 pH units arising from the analysis of EPR titration curves and, therefore, were neglected. After the residual powder was filtered off and compressed between filter papers to remove excess liquid, the sample was placed inside the quartz EPR tube (i.d. = 3.5 mm), and EPR spectra were recorded immediately thereafter. Sealing of the samples inside the quartz tube prevented water evaporation from the pores. Detailed descriptions of EPR titration experiments and analysis of EPR spectra can be found in [26,31,38].

All EPR spectra were recorded at room temperature (≈293 K) using the X-band CW Bruker Elexys E-500 EPR spectrometer. Aqueous solutions of NR were positioned into open-end quartz capillaries with i.d. = 0.50 mm and o.d. = 0.70 mm (VitroCom Inc., Mountain Lakes, NJ, USA), the ends of the capillary were sealed with Critoseal^®^ (Leica Microsystems Inc., Buffalo Grove, IL), and placed in a standard quartz tube (i.d. = 3.5 mm). The tube was consequently inserted into an EPR resonator.

All EPR titrations were carried out in a solution of I = 0.1 M. Errors of the individual measurements for NR in the bulk aqueous phase were ±0.002 mT while for NR confined inside xerogels those values were ±0.004 mT. To record the EPR spectra, the optimal conditions of microwave power and amplitude of modulation of the magnetic field were chosen.

### 4.4. Titration Curves Plotting

Further processing of the experimental spectra was carried out using J. Freed’s software [39] and Microcal OriginPro 2015. The experimental hyperfine coupling constant (HFS) characterized by parameter *a,%* was plotted against *pH_ext_*.

The dissociation constants of the functional groups of the NR used were calculated from the Henderson–Hasselbalch Equation (1) for dibasic acids, as follows:

*a = (P*_1_* + P*_2_* × *10*^pKa1 − pH^ + P*_3_* × *10*^pKa1 + pKa2 − 2pH^)/(*1* + *10*^pKa1 − pH^ + *10*^pKa1 + pKa2 − 2pH^)* (1)

where *P*_1_, *P*_2_, and *P*_3_ are parameters that correspond to the HFS constants of the radical (*a_N_*) in different ionization states of RH_2_^++^, RH^+^, and R, respectively; *pK_a1_*, *pK_a2,_* and *pK_a3_* are the antilogarithms of the ionization constants for RH_2_^++^, RH^+^, and R, respectively; *a* is the HFS constant.

The electrosurface properties were obtained from the analysis of the anisotropic signal of the NR EPR spectra. The change in the fraction of the non-protonated form (*f*) of the NR adsorbed on the surface of the solid-phase material was monitored in the range of *pH_ext_* from 1 to 10, as follows:

*f = n_R_/(n_R_ + n_RH2++_)* (2)

where *n_R_* and *n_RH2++_* are the fractions of slow-motioned radical molecules in the non-protonated form and the protonated form, respectively.

### 4.5. SEP Calculation

In the case of the pH-sensitive probe adsorbed on the surface of the material under study, Khramtsov et al. in [24] established a relationship between the surface potential and the ionization constant of pH-sensitive NR, as follows:

∆*pK^el^= −(e × φ/*2.3*) × k × T* (3)

where ∆*pK_a_^el^* is the shift in *pK_a_* of the NR in the sample induced by an electric potential; *e* is an electron charge; *φ*—electrostatic potential, mV; *k* is the Boltzmann constant; *T* is the temperature, *K*.

## Figures and Tables

**Table 1:** Characteristics of the synthesized binary TiO_2_-SiO_2_ xerogels.

TiO_2_ Content, %	S, m^2^/g	Ratio of the Absorption Band IR Intensities of Ti–O–Si and Si–O–Si Groups at 950 cm^−1^ and 1079 cm^−1^
0	27.8	0
7	254.3	0.5
50	16.4	0.2
100	0.7	0

**Table 2:** Acid–base and electrosurface characteristics of the samples studied.

Sample	*pK_a_* of Functional Groups ± 0.08		∆*pK^el^* ± 0.08	*SEP* ± 6 mV
SiO_2_	5.30	-	2.73	−159
TiO_2_ (7%)-SiO_2_-	5.60	1.90	3.37	−196
TiO_2_ (50%)-SiO_2_-	-	3.20	0.73	−42
SiO_2_ annealed	5.70	-	2.36	−137
TiO_2_ (7%)-SiO_2_- annealed	5.60	2.90	3.50	−204
TiO_2_ (7%)-SiO_2_-Cu(II)	5.30	-	1.97	−115

**Table 3:** The differences in *pH_ext_* and *pH_loc_* for the oxide xerogels.

Sample	∆*pH* *= pH_ext_ − pH_loc_*	
	*pH_ext_* ≤ 4.5	*pH_ex_*_t_ ≥ 4.5
SiO_2_	−0.82	−0.1
TiO_2_ (7%)-SiO_2_	−1.49	−0.37
TiO_2_ (50%)-SiO_2_	−1.21	−0.17
TiO_2_	−0.84	−0.04
